# Managing cataract surgery in patients with glaucoma

**Published:** 2019-02-10

**Authors:** Fatima Kyari

**Affiliations:** 1 Consultant Ophthalmologist Medical Education Coordinator, Baze University, Abuja, Nigeria.

**Cataract and glaucoma can co-exist. Learn about cataract surgery in patients with previous trabeculectomies, and when (and how) to combine cataract and glaucoma surgery.**

**Figure:**
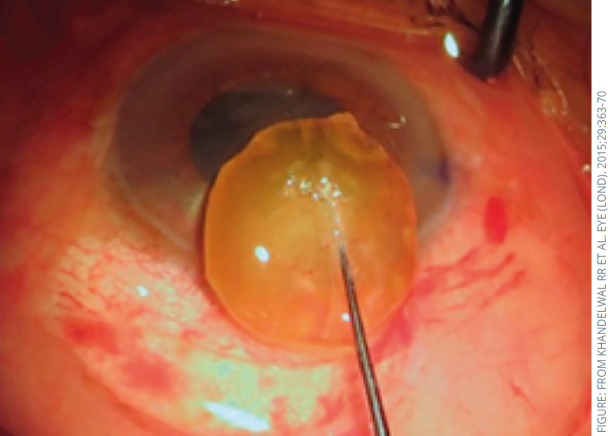
Cataract and glaucoma surgery can sometimes be combined.

Cataract and glaucoma are leading causes of blindness and visual impairment. Both conditions are age-related and thus they may co-exist. Cataracts may also cause glaucoma (phacomorphic glaucoma) and cataract may be accelerated as a result of glaucoma surgery.

When cataract co-exists with glaucoma, cataract may be the trigger for seeking health care because the patient notices the coudy vision and white pupil caused by cataract, whereas the gradual visual loss due to glaucoma often occurs without the patient being aware of it until the glaucoma is in its advanced stages.

Glaucoma medication can increase the negative symptoms of cataract, such as miotics (e.g. pilocarpine), which can make the vision worse, and adrenergics, which can increase glare due to a degree of pupillary dilation.

In a patient with cataract, it is important to assess the optic nerve head to exclude glaucoma. If there is no view of the optic disc, other signs, such as relative afferent pupillary defect (RAPD) and raised intraocular pressure (IOP), may indicate co-existing glaucoma.

Cataract extraction, by whichever method, may be combined with any surgical glaucoma technique, including trabeculectomy, glaucoma drainage devices, minimally invasive glaucoma surgery (MIGS), and laser procedures such as endoscopic cyclophotocoagulation (ECP). **Note:** combined surgery is not recommended in patients with uveitic glaucoma (p. 82).

Performing glaucoma surgery alone may accelerate the cataract, which means the patient will need a cataract operation soon. Performing cataract surgery alone may lower the intraocular pressure (IOP) independently,^1^ but this is inconsistent, especially in poorly controlled glaucoma with severe visual field loss.^2^ There is inconclusive evidence of an increased risk of complications in combined surgery over cataract surgery alone, and information about long-term outcomes (follow-up of five years or more) is not available.^3^^,^^4^

Whether cataract surgery is done after trabeculectomy surgery (at least six months after) or combined with trabeculectomy surgery, the **pre-operative assessment** should include counselling to help patients understand that the visual outcome of their operation will depend on the degree of optic nerve damage they have. Keep their expectations modest. Inform patients about the possibility of **bleb failure**^5^ and that **glaucoma medications** may still be required postoperatively, in the long term.

## Cataract surgery for patients who have had previous trabeculectomy

This is regarded as complicated cataract surgery, requiring thorough preoperative examination and preparation, a surgical plan and proactive postoperative care.^6^^,^^7^

Note the following:

- Visual symptoms (e.g., glare, haloes around lights), **visual acuity**, and the possible causes of reduced vision (due to the contribution of the cataract and/or the progression of glaucoma).
- The **severity of the glaucoma**. Estimate optic disc cupping, which is visible if the lens opacity is not dense. The current IOP will determine target IOP and predict the need for another glaucoma intervention.
- The number and frequency of **glaucoma medications** currently used to control IOP. This may indicate whether the bleb is fully functioning or not.
- The number and type(s) of **previous surgery** and the presence of any drainage device. For recent trabeculectomy or implant, it is advised to delay cataract surgery until the bleb matures (at about six months).
- The **bleb** position will determine the approach for the cataract surgery incision, which should avoid the bleb area. Note whether the bleb is functioning, and whether it is cystic or flat and/or fibrosed or vascularised. This will determine whether intraoperative bleb revision is required.
- The position of the **peripheral iridectomy** and the pupil's ability to dilate. Iris manipulation during cataract surgery with floppy iris or poor dilation due to posterior synechiae may increase the risk of postoperative inflammation, which may compromise bleb function.
- **Pseudoexfoliation**, as this may be associated with weak zonules, which is a reason for caution in cataract surgery.
- **Previous surgery**, which may also cause weak zonules and vitreous disturbance, as well as **corneal endothelial cell loss**, which poses a greater risk of postoperative corneal oedema and decompensation.

Intraoperatively, use extra caution and pay attention to the following:

- **Care of the existing bleb**, especially if thin and cystic, during placement of the lid speculum and any physical manipulation or instrumentation of the eye during wound incision and paracentesis.
- **Bleb revision**, if needed. This can be done using needling and/or additional application of antimetabolites.
- **Maintaining the anterior chamber (AC) depth**, especially in patients with a high functioning bleb. Take extra caution to prevent posterior capsular rent and vitreous loss. Ensure that any tube that is in place is not blocked, obstructed or displaced.
- **Review a functioning tube**, if present. It can be repositioned or flushed to improve the chances of it functioning after surgery. It may also be trimmed if too long.
- **IOL placement.** The aim is to place the IOL in the bag otherwise, be prepared for a sulcus placement if the capsule or zonules are compromised. Position the lens so that the haptic is away from the primary incision (to avoid it migrating into the AC). Avoid AC IOLs because they can directly affect the bleb and may produce undue postoperative inflammation.
- **AC washout.** Thoroughly remove viscoelastic as well as nuclear and cortical lens material. This will prevent blockage of drainage channels and/or tubes and reduce postoperative inflammation and IOP spikes. However, take care to prevent excessive flushing against the corneal endothelium as this can cause damage.
- **Wound closure.** Use sutures to produce a watertight wound. A wound leak will increase the risk of bleb failure.

Proactive postoperative care involves regular visits and long-term follow-up to assess IOP, visual fields, and bleb morphology and function.

- Watch out for **bleb failure** - examine for bleb leak and aqueous misdirection. Actively check for retained lens fragments or iris incarceration in the sclerectomy site that may cause blockage.
- Aggressively treat postoperative inflammation to **prevent fibrosis**. At the end of surgery, give an extra subconjunctival corticosteroid injection (e.g. 4 mg dexamethasone). Avoid miotics and prostaglandins and use more postoperative steroid eye drops (e.g. dexamethasone hourly) and non-steroidal anti-inflammatory eye drops (e.g. diclofenac).
- Measure the IOP regularly and treat **IOP spikes** with appropriate glaucoma medications.

## Combined cataract and glaucoma surgery

Combined cataract and glaucoma surgery may be the procedure of choice if the skill of the surgeon is adequate, the patient understands the procedure and expected outcomes, and there will be good and regular follow-up. Consider combined surgery when:

- There has been no previous glaucoma surgery.
- There is a definite surgical indication for glaucoma surgery, such as angle-closure glaucoma with co-existing cataract.
- Cataract surgery alone is likely to increase the IOP in the patient with glaucoma. This may be the case in patients with poorly controlled glaucoma and/or severe visual field loss.
- It is more cost-efficient to do combined surgery; for example, if there is a one-off payment for surgery and/or a reduction in the number of glaucoma medications used.
- Patients are deemed unlikely to come back for the second operation if the cataract and glaucoma operations are done separately.
- There is an opportunity to provide glaucoma surgery for patients who were initially reluctant until vision loss from cataract influenced their decision to have surgery.
- It is preferable to offer combined surgery because of increased anaesthetic/surgical risks due to other systemic conditions such as hypertension, diabetes, asthma and thyroid disease.

The safe surgery system trabeculectomy combined with cataract extraction (either manual small-incision cataract surgery (MSICS) or phacoemulsification) was developed by Moorfields Eye Hospital. It has been demonstrated to result in excellent IOP control with minimal postoperative complications.^8^ There was a success rate of at least 50% for achieving IOP of ≤12 mmHg, and a success rate of up to 90% for achieving IOP ≤18 mmHg. The technique offers an acceptable option for surgical treatment in patients with cataract and coexisting glaucoma in low- and middle-income countries. Another possible solution in countries where laser facilities are available, is combined cataract surgery with endoscopic cyclophotocoagulation (ECP),^9^ especially where the risks of trabeculectomy with mitomycin C (MMC) are higher (e.g., in eyes with a thin conjunctiva or secondary glaucoma). This may be performed as a two-site procedure or through the same superior incision.

**Figure 1:**
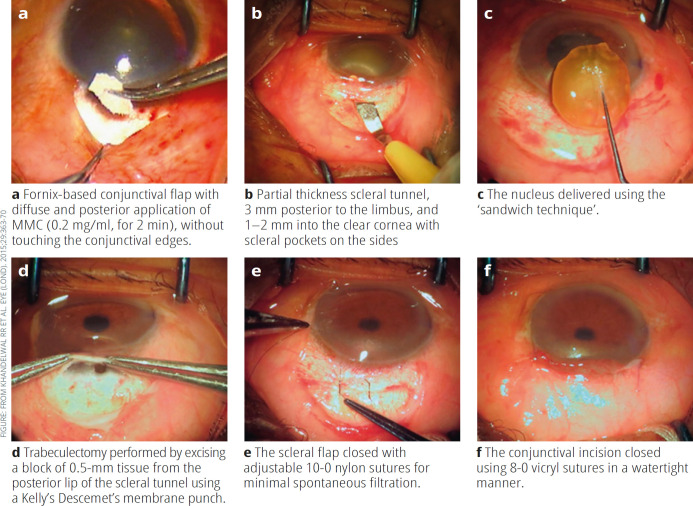
Intraoperative photograph showing surgical procedure of MSICS–trabeculectomy

The steps of the safe surgery system trabeculectomy technique with modifications to combine it with surgery (MSICS), adapted from Khandelwal et al,^8^ are described below and in Figure 1 (a–f).

### A Preparing the scleral area for trabeculectomy

1. Use peribulbar anaesthesia or a retrobulbar block.
2. Clean the periocular area with 5% povidone iodine for 3 minutes.
3. Insert a superior rectus traction suture with 6-0 silk, taking care not to damage the conjunctiva.
4. Dissect a fornix-based conjunctival flap superiorly and separate the Tenon's capsule. Note: a temporal/nasal position may be considered to allow for possible repeat glaucoma surgery.
5. Dissect the conjunctiva backwards for about 8–10 mm to allow for the application of MMC sponges.
6. Insert three MMC (0.2 mg/ml) folded soaked sponges in the sclera, keeping away from the edges of the conjunctiva.
7. Remove the sponges after two minutes and thoroughly irrigate the area with 20 ml Ringers lactate or normal saline solution.

### B Performing the MSICS

1. At about 3 mm posterior to the corneal limbus, use a crescent blade to make a partial thickness scleral tunnel of 5–7 mm in width.
2. Continue the dissection for 1–2 mm into the clear cornea with the scleral pockets continued in the same plane. Leave the sides of the scleral tunnel intact.
3. Make a temporal corneal paracentesis and inject viscoelastic.
4. Perform a capsulorrhexis of about 5–6 mm through the side port.
5. From the scleral tunnel position, enter the anterior chamber with a 3.2 mm keratome at 12 o'clock to enlarge the inner lip to 8-9 mm wide.
6. Using hydrodissection, rotate and prolapse the lens nucleus into the anterior chamber using a Sinskey hook.
7. Deliver the nucleus with an irrigating vectis or with a wire vectis and a Sinskey hook. Use the sandwich technique after injecting generous viscoelastic above and below the lens nucleus.
8. Aspirate the remaining cortex using a two-way irrigation-aspiration simcoe cannula.
9. Insert an IOL, placed in the bag and rotated such that the haptics are positioned horizontally and away from the scleral incision.

### C Performing the trabeculectomy

1. After insertion and positioning of the IOL, aspirate the viscoelastic and inject acetylcholine to constrict the pupil.
2. Excise a block of 0.5 mm tissue from the posterior lip of the scleral tunnel. A Kelly's Descemet's membrane punch or similar device can be used, where available.
3. Follow with a peripheral iridectomy corresponding with the inner scleral flap.
4. Close the scleral tunnel with two adjustable 10-0 monofilament nylon sutures inserted on either side of the punch area. This is the key step for the safe surgery system trabeculectomy. Note: make four throws when tying the suture.
5. For brown and hard nuclei, where the scleral tunnel is enlarged at the sides, insert additional interrupted sutures at the corners of the scleral tunnel.
6. Close the conjunctiva with 10-0 nylon suture using anchoring corneal suture technique to produce a watertight closure under tension.
7. Test the patency of the trabeculectomy by injecting Ringers lactate or normal saline through the side port to observe the formation of a diffuse conjunctival bleb without a leak.
8. Inject subconjunctival dexamethasone and antibiotics in the inferior fornix.

### D Postoperative care

Postoperatively, patients are treated with antibiotic eye drops (e.g. ciprofloxacin 0.3%) four times a day, corticosteroid eye drops (e.g., dexamethasone 0.1% or prednisolone acetate 1 %) six times a day for one week and tapered over two months. Cycloplegics may be used to reduce discomfort. The use of anti-glaucoma medication will depend on the IOP.

The preferred postoperative examination schedule is at postop Day 1, Day 3, at one week, at one month, at 3 months and thereafter scheduled at reasonable intervals, as required. At each visit, the best corrected visual acuity, IOP and complications, if any, are noted.

In addition to applying the basics of good postoperative care after glaucoma surgery,^10^ be alert for the symptoms and signs of complications of cataract surgery.

## References

[1] Masis M Mineault PJ Phan E Lin SC. The role of phacoemulsification in glaucoma therapy: A systematic review and meta-analysis. Surv Ophthalmol. 2018;63:700–710. 28887138 10.1016/j.survophthal.2017.08.006

[2] Bojikian KD Chen PP. Intraocular pressure after phacoemulsification in open-angle glaucoma patients with uncontrolled or marginally controlled glaucoma and/or with severe visual field loss. J Glaucoma. 2018;27:108–114. 29303878 10.1097/IJG.0000000000000854

[3] Zhang M Hirunyachote P Jampel H. Combined surgery versus cataract surgery alone for eyes with cataract and glaucoma. Cochrane Dat Syst Rev 2015;7: CD008671. 10.1002/14651858.CD008671.pub3 PMC4730948 26171900

[4] Mathew RG Parvizi S Murdoch IE. Success of trabeculectomy surgery in relation to cataract surgery: 5-year outcomes. Br J Ophthalmol 2018. [Epub ahead of print] 10.1136/bjophthalmol-2018-312972 30472659

[5] Mathew RG Murdoch IE. The silent enemy: a review of cataract in relation to glaucoma and trabeculectomy surgery. Br J Ophthalmol. 2011 Oct;95(10):1350–4. 21217138 10.1136/bjo.2010.194811

[6] Dada T Bhartiya S Begum Baig N. Cataract surgery in eyes with previous glaucoma surgery: pearls and pitfalls. J Current Glau Prac 2013;7(3):99–105. 10.5005/jp-journals-10008-1145 PMC4741148 26997791

[7] Binder SP. Presentation spotlight: Planning cataract surgery in a patient with previous trabeculectomy. February 2018. Last accessed 1 January 2019.

[8] Khandelwal RR Raje D Rathi A Agashe A Majumdar M Khandelwal R. Surgical outcome of safe surgery system trabeculectomy combined with cataract extraction. Eye (Lond) 2015;29:363–70. 25502867 10.1038/eye.2014.294 PMC4366461

[9] Marco S Damji KF Nazarali S Rudnisky CJ. Cataract and glaucoma surgery: Endoscopic cyclophotocoagulation versus trabeculectomy. Middle East Afr J Ophthalmol 2017;24:177–82. 29422751 10.4103/meajo.MEAJO_232_16 PMC5793448

[10] Kyari F Abdull MM. The basics of good postoperative care after glaucoma surgery. Comm Eye Health 2016;29(94):29–31. PMC5100471 27833261

